# The cost of genetic diagnosis of suspected hereditary pediatric cataracts with whole-exome sequencing from a middle-income country perspective: a mixed costing analysis

**DOI:** 10.1007/s12687-024-00708-9

**Published:** 2024-05-10

**Authors:** Luiza M. Neves, Márcia Pinto, Olivia A. Zin, Daniela P. Cunha, Bruna N. S. Agonigi, Fabiana L. Motta, Leonardo H. F. Gomes, Dafne D. G. Horovitz, Daltro C. Almeida, Jocieli Malacarne, Leticia Guida, Andressa Braga, Adriana Bastos Carvalho, Eduardo Pereira, Ana Paula S. Rodrigues, Juliana M. F. Sallum, Andrea A. Zin, Zilton F. M. Vasconcelos

**Affiliations:** 1 Instituto Fernandes Figueira-Fundação Oswaldo Cruz, Rio de Janeiro, 22250‐020 Brazil; 2 Department of Ophthalmology, Universidade do Estado do Rio de Janeiro, Rio de Janeiro, 20551-030 Brazil; 3 Department of Ophthalmology, Universidade Federal de São Paulo, São Paulo, 04039-032 Brazil; 4 Instituto Brasileiro de Oftalmologia, Rio de Janeiro, 22250-040 Brazil; 5 5Instituto de Genética Ocular, São Paulo, 04552-050 Brazil; 6 Instituto Nacional de Cardiologia, Rio de Janeiro, 22240-006 Brazil; 7 INSEAD, Fontainebleau, France; 8 Instituto Catarata Infantil, Rio de Janeiro, 22250-040 Brazil; 9 Instituto de Biofísica Carlos Chagas Filho, Universidade Federal do Rio de Janeiro, Rio de Janeiro, 21941-971 Brazil

**Keywords:** Whole-exome sequencing, Microcosting, Mixed costing health economics, Congenital cataracts, Pediatric cataracts, Hereditary cataracts

## Abstract

Up to 25% of pediatric cataract cases are inherited. There is sparse information in the literature regarding the cost of whole-exome sequencing (WES) for suspected hereditary pediatric cataracts. Molecular diagnosis of suspected hereditary pediatric cataracts is important for comprehensive genetic counseling. We performed a partial economic evaluation with a mixed costing analysis, using reimbursement data and microcosting approach with a bottom-up technique to estimate the cost of using WES for genetic diagnosis of suspected hereditary pediatric cataracts from the perspective of the Brazilian governmental health care system. One hundred and ten participants from twenty-nine families in Rio de Janeiro (RJ) were included. Costs of consumables, staff and equipment were calculated. Two scenarios were created: (1) The reference scenario included patients from RJ with suspected hereditary pediatric cataracts plus two family members. (2) The alternative scenario considered other genetic diseases, resulting in 5,280 exams per month. Sensitivity analysis was also performed. In the reference scenario, the total cost per exam was 700.09 United States dollars (USD), and in the alternative scenario, the total cost was 559.23 USD. The cost of WES alone was 527.85 USD in the reference scenario and 386.98 USD in the alternative scenario. Sensitivity analysis revealed that the largest costs were associated with consumables in both scenarios. Economic evaluations can help inform policy decisions, especially in middle-income countries such as Brazil.

**Supplementary Information:**

The online version contains supplementary material available at 10.1007/s12687-024-00708-9.

## Introduction

Cataracts are the opacification of the eye’s intraocular lens, resulting in reduced visual acuity. They can affect children at birth or during infancy and lead to serious visual impairment if not appropriately treated with surgery in a timely manner. Pediatric cataracts can be isolated or associated with other ocular abnormalities, multisystem genetic conditions, chromosome disorders or metabolic disorders [1].

Despite being a rare disease with a worldwide [2] and a Brazilian [3] prevalence of approximately 4/ 10,000 children, congenital cataract and others lens disorders represent up to 21.3% of all cases of vision impairment in children globally, although this proportion may be overstated [4]. In Brazil, congenital cataract represents between 6 and 14% of cases of vision impairment in children from low vision schools [5, 6].

Brazil is a middle-income country with a large and publicly funded health care system (SUS) that is universally available and used by more than 70% of its population (approximately 160 million) [7]. Brazil has a “National Policy for Comprehensive Care of People Affected by Rare Diseases within SUS’’, which has promoted health care equity for these patients since 2014. It addresses comprehensive and multidisciplinary care, including diagnosis, genetic counseling, treatment and rehabilitation [8]. Although 80% of rare diseases are genetic and 33 genetic services exist in SUS, it is estimated that only half of these of services perform molecular biology techniques for genetic investigation [9].

Comprehensive genomic diagnostic tests such as targeted panels, whole-exome sequencing (WES) and whole-genome sequencing (WGS) have improved our knowledge of genetic diseases and have enhanced access to personalized medicine [10]. These methods are recommended for genetic diseases with a significant overlap in clinical presentation or for which there are many potential genes related to a specific clinical presentation. WES is a molecular test that identifies variants in all protein-coding regions, although there are limitations, such as the inability to detect deep intronic sequences or structural variants, when compared to WGS [10]. Usually, WES is more available than WGS due to its lower cost [10].

Currently, 503 genes are associated with pediatric cataracts [11]. Identifying the molecular causes of pediatric cataracts is important because between 8.3 and 25% of cataracts are inherited and 15% are associated with a systemic disease, where the eye can be a sentinel organ [1].

Ten years ago, the World Health Organization recommended measures to implement DNA-based diagnosis in low- and middle-income countries to increase their expertise in genomics [12]. These recommendations include training services, public education, development of bioinformatics and bioethics, and promotion of research resource allocation [12, 13]. Despite these efforts, an enormous gap among low-, middle- and high-income countries still remains. For example, less than 10% of genetic laboratories registered by the *Genetic Testing Registry* are located in middle-income countries, while more than 90% are located in high-income countries [14].

Following advances in the field of genetics, the Brazilian government launched the National Genomic Program in 2020 to embed precision medicine in our public health care system. The main objective was to create a national genomic database with 100,000 Brazilian genomes, including those of patients with rare diseases, cardiopathies, cancers and infectious diseases [15]. However, obtaining a molecular diagnosis is challenging for Brazilian patients with diseases that might have a genetic etiology, such as pediatric cataracts. First, genetic testing is not widely available within SUS; it is mostly available in specific rare disease reference centers in the southeastern region of the country and only for a few specific diseases [9, 16]. Second, there is a bottleneck for accessing reference centers, and rare disease patients may wait up to one year for a genetic consultation within SUS [9, 16]. The cost of genetic testing for pediatric cataracts is believed to be another major obstacle considering the middle-income status of the country [17].

Technical performance and clinical indications for genetic testing have been extensively discussed in the literature, but economic evaluation has emerged asfundamental way to obtain information for multidisciplinary decision-making in health care [18, 19]. In Brazil, the National Commission of Technology Incorporation recommends the incorporation of technologies within SUS based on economic evaluation studies. Cost analysis, such as that performed in this study, can be a substrate for future complete economic evaluations [20].

The purpose of this study was to perform a cost estimation of genetic diagnosis via WES for suspected hereditary pediatric cataracts through the perspective of the Brazilian national health care system. Since genetic diagnosis is possible only after the clinical confirmation of pediatric cataracts, we sought to estimate the cost of both clinical and genetic diagnosis.

## Materials and methods

### Brazilian context

There are 27 reference centers for the management of patients with rare diseases in Brazil, and the *Instituto Nacional de Saúde da Mulher, da Criança e do Adolescente Fernandes Figueira (IFF)* in Rio de Janeiro is one of them. Patients with rare eye diseases are managed in ophthalmic centers, and those with a suspected genetic etiology are referred to rare disease reference centers. However, it is estimated that only 30% of all patients with rare diseases requiring clinical evaluation by specialized professionals receive an evaluation, representing a bottleneck toward the final genetic diagnosis of patients with rare eye diseases, such as pediatric cataracts [16]. In this scenario, the government pays up to 620 USD per year for the management (diagnosis, treatment, and rehabilitation) of a patient with any rare disease [21].

The *Instituto Nacional do Coração* (INC) in Rio de Janeiro is conducting an ongoing national research project for the diagnosis of suspected hereditary cardiovascular diseases (The Brazilian National Network of Cardiovascular Genomics) [22] and, in addition, performs molecular diagnoses of other genetic diseases. The INC and IFF established a partnership for testing for genetic diseases, where the IFF sends DNA samples from patients with rare diseases for next-generation sequencing to the INC.

### Study design and perspective

This study is a cost analysis study of suspected hereditary pediatric cataract genetic diagnosis through the SUS perspective. We performed a mixed costing analysis, using reimbursement data to estimate the cost of clinical diagnosis and a microcosting approach with a bottom-up technique to estimate the cost of genetic diagnosis. The latter approach was used because genetic testing via WES for ocular diseases is not currently available in clinical practice in the SUS. The bottom-up technique uses detailed activity and input usage data from records or from observation (as in this study) at a health care service to estimate unit costs [23]. In our case, we obtained data from the observation of a sample of suspected hereditary pediatric patients who underwent WES. The analysis was performed using Excel 365 software (Microsoft, USA).

### Population and setting

The eligible population included all patients with pediatric cataracts up to 18 years old in Rio de Janeiro and two additional family members, as our study included trio analysis for WES. The population estimate included epidemiological incidence and prevalence data for the first year of the economic model and incidence data for each of the 4 subsequent years, given the 5-year time horizon. We included a pediatric cataract cumulative incidence of 3.46:10,000 for the 15-year interval [24] and a congenital cataract incidence of 4:10,000 [3]. We assumed that the prevalence of pediatric cataracts would be the same as that of congenital cataracts, and we assumed that the cumulative incidence for 15 years would remain the same up to 18 years of age. We considered that 76% of the population uses the SUS, based on official governmental data from Brazil [25].

To identify and quantify health resources, a sample of patients with familial pediatric cataracts from the CATBRA study was used. The CATBRA study, a Brazilian study for genomic evaluation of suspected hereditary pediatric cataract patients (protocol code 21444619.0.0000.5269), aims to identify variants associated with suspected hereditary pediatric cataracts, to analyze the impact of the disease on the management of these patients and families, and to perform a budget impact analysis of WES for pediatric cataracts. The eligible population of the CATBRA study included one hundred and ten participants from twenty-nine different families within a cohort of pediatric cataract patients from a nonprofit health organization dedicated to the management (diagnosis, treatment and follow-up) of pediatric cataracts in Rio de Janeiro, Brazil [26]. The inclusion criteria were patients up to 18 years old with a history of pediatric familial cataracts in any family member, such as parents, siblings, grandparents, uncles or cousins. The exclusion criteria included a history of congenital TORCH infections (toxoplasmosis, rubella, cytomegalovirus, herpes simplex, syphilis, varicella zoster, or Zika) and the use of corticosteroids or ocular trauma.

This study was justified by the opportunity to carry out a cost analysis nested within the aforementioned study and by the possibility of identifying the causative variants of pediatric cataracts in the same family.

We considered pediatric cataracts to include those that appeared at birth (congenital cataracts) and those that appeared during infancy (childhood or infantile cataracts).

### Technology

**Fig. 1:**
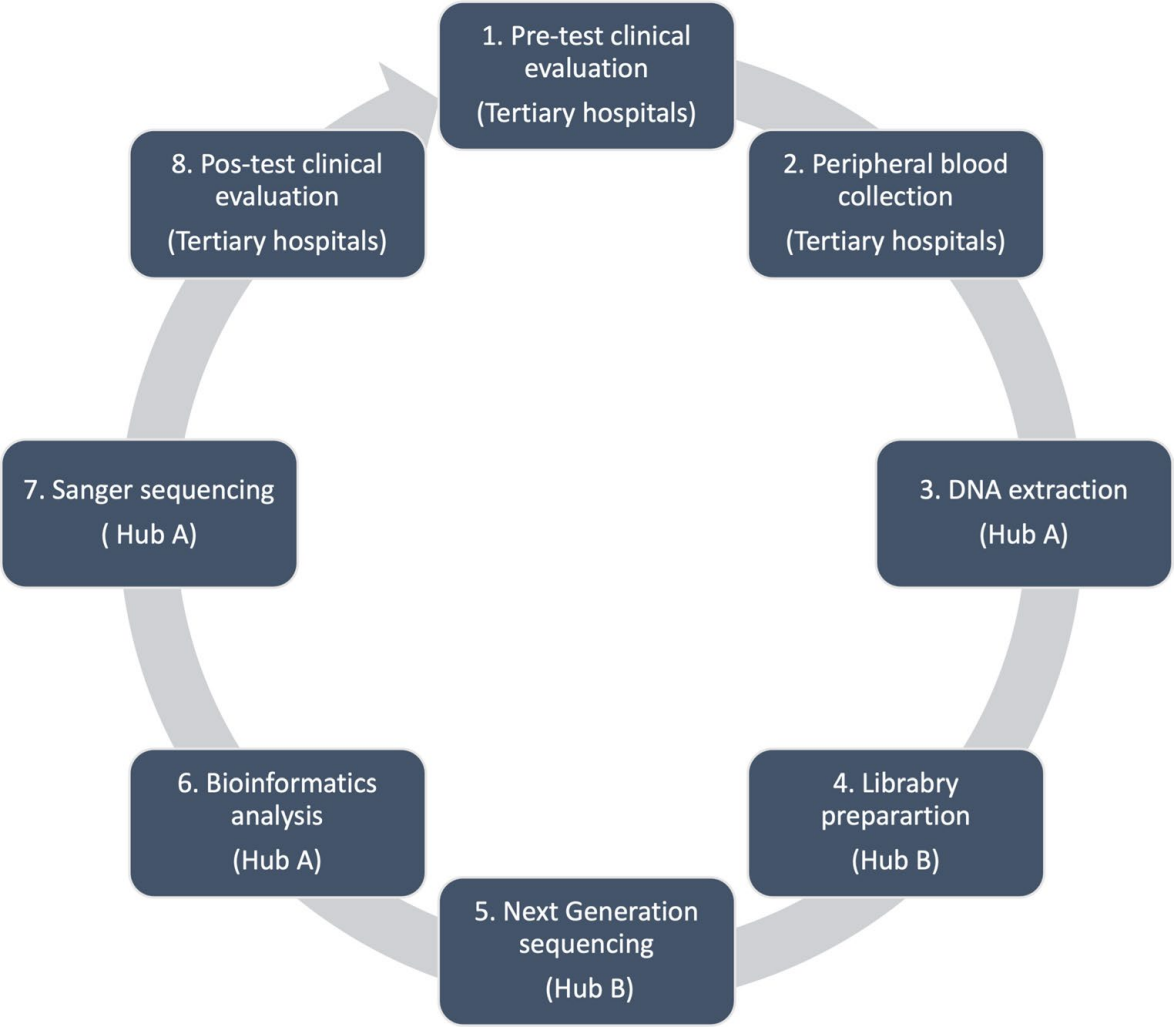
Diagnosis protocol for pediatric cataract patients comprising 8 steps: pre-test evaluation, peripheral blood collection, transportation of biological material, DNA extraction, library preparation, next-generation exome sequencing, bioinformatic analysis, Sanger sequencing, and post-test clinical evaluation

**Fig. 2:**
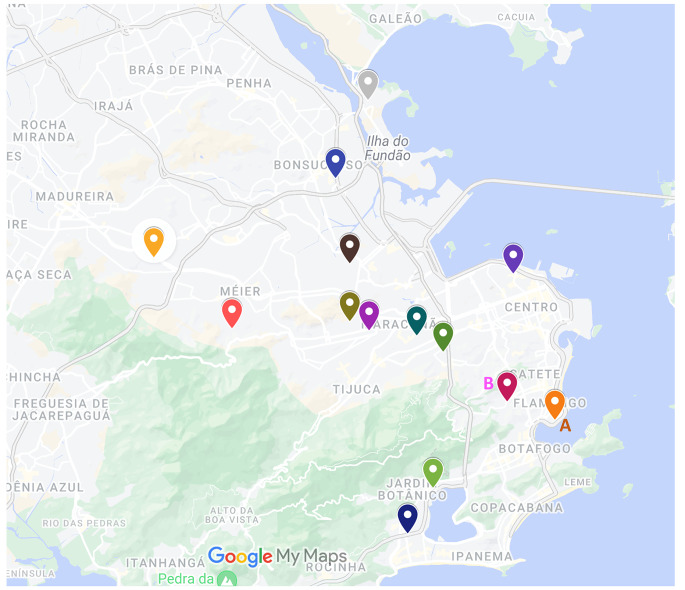
Twelve governmental tertiary hospitals with pediatric cataract patients eligible for ocular genetic testing in the city of Rio de Janeiro (Fig. 2). Hospitals A and B were selected as hubs

The genetic diagnosis of pediatric cataracts via WES was evaluated. The diagnostic protocol was divided into 8 steps to estimate the cost of each step (Fig. 1). A theoretical network of the 12 main governmental tertiary hospitals in the city of Rio de Janeiro with pediatric cataract patients eligible for ocular genetic testing was created, and two facilities (A and B) responsible for genetic testing were selected as hubs (Fig. 2). The steps were as follows:

1. The pre-test clinical evaluation included two clinical ophthalmologic consultations and one clinical genetic consultation. The clinical diagnosis process included ophthalmic exams (slit-lamp biomicroscopy, intraocular pressure, indirect ophthalmoscopy, ocular ultrasonography and biometry for axial length measurement) and clinical genetic evaluations (family history, origins and pedigree; existence of other systemic conditions; and clinical‐morphological evaluation) [27].
2. Peripheral blood sample collection from eligible patients with pediatric cataracts and their family members was performed at each of the 12 tertiary hospitals (Fig. 2).
3. Genomic DNA extraction and evaluation of the quantity and quality of the genomic DNA from the peripheral blood sample were performed at hospital A (IFF).
4. Library preparation refers to the preparation of DNA for high-throughput sequencing of the exome, which was performed at facility B (INC).
5. WES, which involves sequencing of the protein-coding regions of the genome, was performed at facility B (INC).
6. Bioinformatics analysis included the use of computational tools and techniques for remote interpretation of WES data by an expert team.
7. Sanger sequencing for the validation of suspected pathogenic variants was performed in facility A (IFF).
8. The post-test clinical consultation included one clinical genetic consultation for genetic counseling and care at tertiary hospitals (Fig. 1).

Additionally, we estimated the operational cost of this process, which included administrative and logistic functions and biologic material transportation.

### Cost analysis scenarios

We estimated the cost of genetic diagnosis of pediatric cataracts in two scenarios. The first scenario, our reference scenario, included patients with pediatric cataracts and two family members (one affected and one unaffected) in the city of Rio de Janeiro. We assumed that trio analysis was needed for WES.

The second scenario, an alternative scenario, was created to estimate the minimal cost of WES with the hub working with maximum capacity. Currently, hospital B (INC), which sequences multiple cardiac genetic diseases, such as hypertrophic cardiomyopathy, within the research budget, has an Illumina NovaSeq 6000 sequencer [22]. Like for pediatric cataract cases, there is no reimbursement expected for genetic testing for cardiac diseases within the SUS [22]. Given that the sequencer has an annual capacity (63,360) of almost eight times the annual pediatric cataract demand in Rio de Janeiro (approximately 8,000), it should not only be used for pediatric cataracts but also for sequencing patients with other genetic diseases. The monthly capacity of the sequencer was determined based on the following characteristics: 2 × 100 bp coverage with two S4 flowcell plates with 240 samples each run for 36–48 h, 22 days per month ([240 samples per plate × 2 plates × 22 workdays in a month]/2 days, which is the longest duration of sequencing).

### Costing model

Following the Brazilian Guideline for Budget Impact Analysis [28], we considered a five-year time horizon, with the first year costs including the acquisition and maintenance costs of the equipment plus consumables and staff and the second, third, fourth, and fifth years including consumables, staff and equipment maintenance costs.

The costs of pre-test clinical evaluation (step 1), post-test clinical evaluation (step 8) and complementary exams, such as ocular ultrasonography and biometry (step 1), were based on the SUS reimbursement table of ambulatory procedures [21]. This table provides the total reimbursement cost of the procedure, including consumables, staff and equipment costs.

The costs of the other steps are not provided by the above mentioned table and were estimated through the microcosting technique [21]. Peripheral blood sample collection (step 2), genomic DNA extraction and evaluation of genomic DNA quantity and quality (step 3), library preparation (step 4), exome sequencing (step 5), bioinformatics analysis (step 6), Sanger sequencing (step 7) and operation were estimated based on direct observation of the genetic testing performed in the one hundred and ten participants of the study, enabling us to list all the resources used.

Every step of the genetic testing protocol was parsed into an Excel spreadsheet to identify, quantify, and value each item used in the procedure. The cost of each step included the costs of disposable consumables, equipment and hands-on staff time. If available, components were priced using an official database [29]. Otherwise, the products were priced according to the manufacturer or wholesale supplier (Thermo Fisher Scientific, Sigma‒Aldrich, Illumina, Promega, Eppendorf). Overhead costs, including water, cleaning, electricity, safety, the internet, food and administrative items, were assumed to constitute a total of 10% of the cost per exam and were included in the operational step costs. Usually, overhead costs range between 20 and 30%; we assumed the lower rate because the facility’s infrastructure already existed.

Consumable costs were reduced to a per-unit cost (per exam). To estimate each step’s cost, the necessary item cost per unit was listed and multiplied by the minimal batch quantity needed for the eligible population. Given that reagent kits and some disposable consumables are available only in batches of a minimal quantity, if there is one additional patient above the minimal batch quantity, it will be necessary to buy another batch.

To estimate the equipment cost, we listed all the equipment used and distributed it between the two main facilities (A and B) according to the above steps. We assumed that the Illumina NovaSeq 6000 sequencer (facility B - INC) would work well, analyzing 5,280 samples monthly, as explained in the ‘scenarios section’, in both the reference and alternative scenarios. For other equipment, we estimated the quantity needed to perform 5,280 samples monthly, assuming maximum performance in a 40-hours/week workload, for 22 days per month (supplemental material). Equipment was priced according to the manufacturer or wholesale supplier. We did not use a straight-line depreciation rate because it is not included in the Brazilian Guidelines [28]. We considered the acquisition cost in the first year, and the maintenance cost in every year. Annual maintenance was estimated at 5% of the equipment cost [30].

To estimate the cost per step if one piece of equipment from the same facility was used in more than one step, we used a pro rata distribution to adjust its cost by the equipment usage percentage compared to the other steps. For instance, the Thermo Fisher QUIBIT is used in steps 3, 4, 5 and 7 but is used more frequently in step 5. Therefore, we assumed that its percentage of use in these steps would be 20%, 20%, 40% and 20%, respectively. In contrast, NanoDrop 8 is used in steps 3 and 7; we assumed its percentage of use as 50% for each step. The distribution is available in the supplementary material.

The annual salary of each personnel job class was obtained from hospital A, as this ward was responsible for the main steps. To obtain the real annual salary, the monthly wage was multiplied by 14.3 in accordance with the Brazilian Labor Law. This adjustment included 1/3 of the monthly salary for vacation, a thirteenth of the salary for December and a one-month payment for a staff substitute during mandatory vacation. We assumed a 40 h per week workload and 52 weeks per year. The personnel performed the specific step for 6 h a day, with the remaining 2 h for computer work and lunchtime. We estimated weekly, monthly and annual productivity per personnel according to the hands-on time per step.

If a suspected pathogenic variant was found during WES in CATBRA study, we performed Sanger sequencing for the single gene. With respect to the model, we assumed that half of the patients would need Sanger sequencing.

The logistic cost included the cost of transporting patient blood samples from tertiary hospitals to hub hospitals. Hospital A (IFF) received the samples to perform DNA extraction, library preparation and, after WES, Sanger sequencing for confirmation of pathogenic variants. Next, hospital B (INC) received the products for library construction and WES.

In our reference scenario considering pediatric cataract patients, we assumed the frequency of transportation based on the activity of each pediatric ophthalmology department. Hospitals with lower activity would need transportation once every 3 months; hospitals with moderate activity would need transportation once a month; and hospitals with high activity would need transportation twice a month. The Google Maps platform was used to calculate the distance among the units, assuming a car performance of 8 km per liter for the gasoline cost estimator. We also assumed that it would be possible to use one of the available vehicles at hospital A. Staff included one hired driver for these periods.

We considered the exchange rate to be 1.00 USD = R$5,1686 (from January 2022-June 2023) [31]. Model data are available upon request.

### Outcomes

The primary outcome was the cost per genetic of diagnosis of suspected hereditary pediatric cataracts via WES. The secondary outcome was the cost per step.

### Ethics

The study was approved by the Ethics Committee of INSTITUTO FERNANDES FIGUEIRA-IFF/FIOCRUZ‐RJ/MS (protocol code 21444619.0.0000.5269), 17 October 2019.

### Sensitivity analysis

We conducted a one-way sensitivity analysis for fluctuations in the unit costs of the most expensive consumables (+ 30% and − 30%), staff (+ 30% and − 30%) and equipment (+ 100% and − 20%). The choice of these range values was justified by the absence of studies estimating the cost of WES for familial pediatric cataracts within the SUS, so we used range values from another ocular disease [30, 32]. As in our reference and alternative scenarios, the equipment costs were estimated considering the high capacity of the equipment, and we also estimated the cost of genetic testing if all the equipment was used only for pediatric cataract patients and their families. In addition, we performed sensitivity analysis using an overhead range of 20–30%.

## Results

Table 1 shows the following costs per patient for the reference and alternative scenarios, considering the five-year horizon: average cost for genetic diagnosis of suspected hereditary pediatric cataracts, pre-test clinical consultation, peripheral blood sample collection, genomic DNA extraction, library preparation, WES, bioinformatic analysis, Sanger sequencing, operation cost, and post-test cost. Considering only WES, the cost per exam was 527.85 United States dollars (USD) (R$2.728,24) for the reference scenario and 386.98 USD (R$2.000,14) for the alternative scenario. For the reference scenario, consumables represented 62% of the genetic testing cost, followed by 22% for staff, 7% for equipment and the other 9% for overhead cost. In the alternative scenario, consumables represented 84%, staff 5% and equipment 2% of the cost per exam; the overhead cost represented the remaining 9%. The operational cost was 8.61 USD per km driven.

**Table 1:** Cost of pretest and posttest clinical evaluations and costs per step of whole-exome sequencing of hereditary pediatric cataract patients. Costs of whole-exome sequencing discriminated by consumables, equipment, and staff in the reference and alternative scenarios

	Pretest Evaluation	Peripheral Blood Collection	DNA Extraction	Library	NGS	Analysis	Sanger Sequencing	Posttest Evaluation	Operation	Total
*Reference Scenario*										
*Genetic Testing*										
Consumables (Genetic Testing)	-	USD 6.67	USD 16.35	USD 139.22	USD 118.30	USD 0.17	USD 42.96	-	USD 4.70	USD 328.38
Staff (Genetic Testing)	-	USD 4.76	USD 4.25	USD 0.11	USD 0.11	USD 75.56	USD 8.49	-	USD 21.83	USD 115.11
Equipment (Genetic Testing)	-	USD 0.11	USD 2.80	USD 2.86	USD 3.93	USD 0.55	USD 26.12	-	-	USD 36.37
Overhead	-	USD 1.15	USD 2.34	USD 14.22	USD 12.23	USD 7.63	USD 7.76		USD 2.65	USD 47.99
Total Genetic Testing Cost per Exam	-	USD 12.69	USD 25.74	USD 156.41	USD 134.57	USD 83.91	USD 85.33	-	USD 29.19	USD 527.85
Clinical Evaluation	USD 104.53	-	-	-	-	-	-	USD 67.72	-	USD 172.25
Total Cost per Exam	USD 104.53	USD 12.69	USD 25.74	USD 156.41	USD 134.57	USD 83.91	USD 85.33	USD 67.72	USD 29.19	USD 700.09
*Alternative Scenario*										
*Genetic Testing*										
Consumables (Genetic Testing)	-	USD 6.67	USD 16.35	USD 139.22	USD 118.30	USD 0.00	USD 42.96	-	USD 1.77	USD 325.29
Staff (Genetic Testing)	-	USD 1.23	USD 0.87	USD 0.11	USD 0.11	USD 15.76	USD 0.22	-	USD 0.56	USD 18.86
Equipment * (Genetic Testing)	-	USD 0.01	USD 0.08	USD 2.86	USD 3.93	USD 0.08	USD 0.69	-	-	USD 7.65
Overhead	-	USD 0.79	USD 1.73	USD 14.22	USD 12.23	USD 1.58	USD 4.39	-	USD 0.23	USD 35.18
Total Genetic Testing Cost per Exam	-	USD 8.70	USD 19.04	USD 156.42	USD 134.58	USD 17.43	USD 48.25	-	USD 2.56	USD 386.98
Clinical Evaluation	USD 104.53	-	-	-	-	-	-	USD 67.72	-	USD 172.25
Total Cost per Exam	USD 104.53	USD 8.70	USD 19.04	USD 156.42	USD 134.58	USD 17.43	USD 48.25	USD 67.72	USD 2.56	USD 559.23

**Fig. 3:**
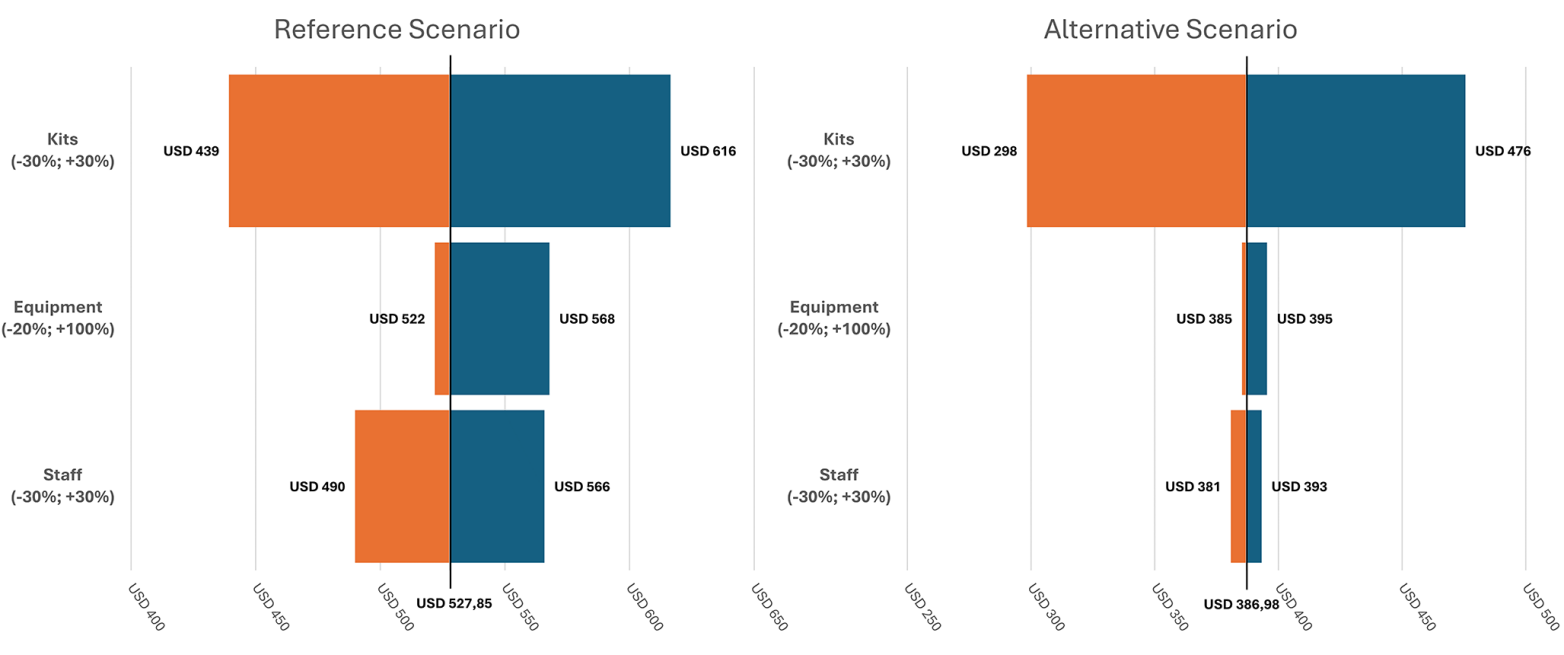
Tornado diagram of parameters (equipment, staff and consumables) impacting the costs per exam in the reference and alternative scenarios. The X-axis represents the impact on costs per exam in United States dollars (USD) of each parameter variation, and the Y-axis represents baseline parameters, with the costs of whole-exome sequencing per exam being 527.85 USD (reference scenario) and 386.98 USD (alternative scenario). The orange and blue bands show high and low values, respectively, of each parameter. The number next to each band corresponds to the cost per exam of whole-exome sequencing in U.S. dollars for that parameter after the sensitivity analysis

Sensitivity analysis revealed that the highest costs in the reference and alternative scenarios were for consumables (Fig. 3). Assuming a 30% increase in consumable costs, the cost per exam increased 17% in the reference scenario and 23% in the alternative scenario. On the other hand, even when the equipment costs doubled, the costs per exam increased by only 7% in the reference scenario and 2% in the alternative scenario. When considering a very low capacity of the equipment, i.e., using it only for pediatric cataract patients and their family members in Rio de Janeiro and not for other diseases, the cost per exam of WES would be 815.28 USD (R$ 4,213.87), 40% from consumables, 36% from equipment and 15% from staff. Using an overhead range of 20–30%, the cost per exam of WES would be 575.83 - 623.82 USD (R$ 2.976,23 - R$ 3.224,27) in the reference scenario and 422.16 - 457.34 ( R$ 2.181,97 - R$ 2.363,80) USD in the alternative scenario.

## Discussion

To the best of our knowledge, this is the first study to estimate the costs of WES for suspected hereditary pediatric cataracts from a middle-income country perspective. Accurately estimating WES costs can be difficult due to the number of steps involved, variations in available resources, differences in researcher methods or preferences, and organization of the health care network. Nevertheless, estimating these costs is a major step in planning resource allocation in any sustainable health care system, particularly in limited-resource settings [17, 33, 34].

It can be argued that genetic testing of pediatric cataract patients should not be a priority because diagnosis is clinical, and surgical treatment does not depend on variant identification. Although these two statements may be true, they should be considered within a broader scenario. Genetic testing is important since up to 25% of pediatric cataracts are inherited [1]. The identification of the causative variant and its pattern of inheritance are fundamental to improving genetic counseling and community education. In our cohort, 13 out of 29 families had three or more affected members who could benefit from precise genetic counseling and genetic follow-up. In addition, 15% of pediatric cataracts are a manifestation of systemic disease, and cataracts may be one of the first clinical findings, as observed in galactosemia and Lowe or Alport syndrom**es** [1].

In the United Kingdom, which has a national health care system similar to that of Brazil, genetic testing is indicated for all bilateral cases of pediatric cataracts [35]. In our cohort, 77% of the suspected hereditary pediatric cataract cases were bilateral. Lenassi et al. showed that genetic testing of pediatric patients with bilateral cataracts avoided unnecessary tests in 50% of those patients [36]. For example, one patient from our cohort underwent an unnecessary phlebotomy and had prolonged use of an oral iron chelator for hyperferritinemia; this patient was subsequently diagnosed with hereditary hyperferritinemia-cataract syndrome after genetic testing [37].

Another benefit of genetic testing is that variant identification in affected families reduces anxiety and enables validation of a clinical diagnosis as well as a feeling of closure for having a final diagnosis, especially for diseases that have no treatment [38], but this testing also benefits patients with disease that can be treated, such as pediatric cataracts.

Questions regarding the affordability of pediatric cataract genetic diagnosis have been raised, but there is scarce information about its cost. A recent systematic analysis of microcosting estimation for genome and exome sequencing did not include any data for pediatric cataract patients [39]. In addition, no data from middle-income countries were included [39], suggesting an uneven geographic availability of genetic cost information.

In the reference scenario, we estimated that the costs of genetic diagnosis for pediatric cataracts were 700.09 USD (R$ 3.618,48) per patient from the SUS perspective. This estimation included pre-test and post-test consultations and complementary exams. Considering only genetic testing with WES, the cost was 527.85 USD (R$ 2.728,24) per patient. As a comparison with other health care technologies in SUS, the cost of microarray genetic testing for intellectual disability is 155 USD (R$ 801,13) [40] while the cost of ocular ultrasonography, used for clinical diagnosis of pediatric cataracts, is 33 USD (R$ 170,56) [21]; .

In the literature, there is considerable variation among microcosting estimates for different diseases [17, 39]. The estimated cost of rare diseases ranged from 993 USD (R$ 5.132,41) per patient for germline mutations to 3,388 USD (R$ 17.511,21) per patient for neurodevelopmental disorders of unknown genetic etiology in high-income countries [39]. Each disease has a specific strategy for analysis, which impacts the genetic testing cost. Nevertheless, consumables for library preparation and sequencing were proportionally responsible for most of the costs, as in our study [41]. Even in the scenario, when we doubled the equipment acquisition cost in the sensitivity analysis, the share of WES costs for consumables decreased from 62 to 58%. In addition, in the worst-case scenario, when the equipment was used only for pediatric cataract patients, the consumables remained the most expensive (40% of the total cost per exam).

Interestingly, the relative cost of WES is likely to depend on the diagnostic yield [42]. This cost is calculated by dividing the per-patient cost of delivering molecular testing by its diagnostic yield. In our reference scenario, the cost per positive diagnosis would be approximately 851.37 USD (R$ 4,400.39) considering the current diagnostic yield for suspected hereditary pediatric cataracts in our institution of 62%. When taking into account a higher diagnostic yield, for instance, 80% for inherited retinal dystrophies [43], the cost per positive diagnosis would be 659.81 USD (R$ 3,410.29). A lower cost per positive diagnosis was achieved with greater numbers of conclusive variants found, more expertise gained, and greater clinical utility of the test over time. In contrast, when testing for a greater number of heterogeneous genetic diseases, as in our alternative scenario, we can hypothesize that there could be a lower diagnostic yield and a greater cost per positive diagnosis.

The prevalence of congenital cataracts is estimated to be 4:10,000 newborns [3], and for that reason, congenital pediatric cataracts is a rare disease according to the definition of the Brazilian Ministry of Health. Despite this, patients with suspected hereditary pediatric cataracts and other rare genetic eye diseases are cared for in ophthalmological services, and genetic diagnosis is still challenging. Genetic testing is available at specific rare disease centers, and it has been estimated that more than 50% of patients with rare diseases do not have access to them [9, 16]. As a consequence, pediatric cataract patients face many obstacles in obtaining a molecular diagnosis. Furthermore, there is no expected reimbursement value for performing WES within the SUS’s clinical practice [44]. The Brazilian federal government pays up to approximately 620 USD (R$ 3,204.53) per patient with a rare disease per year, which includes costs of diagnosis, treatment and rehabilitation [21]. If WES is necessary—and available—it should be included in this budget. Considering our estimation of 527.85 USD (R$ 2,728.24) per WES for pediatric cataracts in the base scenario, the reimbursement for rare disease patients might be underestimated. However, since one of the main principles of SUS is the decentralization of health care assistance, municipalities and states should also contribute to health care financing [45].

The evolving nature of high-throughput next generating sequencing with improvements in sequencing chemistry and technology, as well as bioinformatics and data interpretation, has enabled a greater volume of testing in a shorter time. Additionally, higher-resolution diagnoses are expected to reduce diagnostic and treatment difficulties [33]. Given that, we also created an alternative scenario in which the hub would perform approximately 5,200 analyses (WES, targeted sequencing) per month, including rare and prevalent diseases [22]. Different scenarios allow decision-makers to understand the potential cost implications of resource allocation strategies. In this alternative scenario, the cost per exam would be 386.98 USD (R$ 2,000.14). This means that performing the test on a large scale not only improves patient affordability by decreasing the cost per exam by 27% but also increases patient access to tailored medicine. From the SUS standpoint, other patients with genetic diseases will also benefit from this equipment. As in our reference and alternative scenarios, developing a hub system may be the solution to enhancing access and gain efficiency while reducing the cost per exam. Familial dyslipidemia and genetic cardiomyopathies are among the many common clinical indications for sequencing in our current hub [22].

Brazil is a continental-size country with 160,000,000 people depending on SUS. Elucidating and obtaining the cost of each step of an exam can help policy-makers construct a roadmap for the rational utilization of WES. Additionally, the capillarity of SUS’s network—which is the broad distribution of primary health care services across Brazil, for instance, as high as 40,000 basic units across the country [46]—could be a key element in helping all Brazilians who could benefit from WES. It is crucial to develop a national genomic industrial framework to avoid the risk of being technologically disadvantaged compared to other countries [33].

Our study has several limitations that should be noted. First, we used a microcosting estimation technique, which is often nongeneralized because it is specific for each setting, although it is an accurate and discriminating technique. Second, imported equipment and consumables costs may vary due to exchange rates, especially in low- and middle-income countries. Usually, larger orders allow for better negotiations with suppliers, decreasing the cost of consumables. Third, we performed the estimation in one setting, the Rio de Janeiro municipality, and for one disease, pediatric familial cataracts. However, while the genetic knowledge and diagnostic strategy for each genetic disease differ, the basic technological resources for molecular testing are shared, thus providing an opportunity for extrapolations to other diseases.

There are several remaining challenges associated with incorporating genetic testing for pediatric cataracts in the clinical practice of middle-income countries. The need to keep up with the exponential advances of these technologies and bioinformatic pipelines is one worth mentioning. Additionally, a shortage of experienced professionals, such as bioinformaticians, can be a major barrier, although it can also be an opportunity to improve training and expertise. Concerns regarding data privacy and incidental findings are common challenges expected in any genetic diagnostic scenario, and there should be protocols to guide the team on how to proceed. Patients’ clinical pathways and referrals need to be redesigned to include the test in routine clinical practice. A genetic network would have to deliver timely results to ensure the best clinical outcome and efficiency. Other studies focusing on clinical utility, cost-effectiveness, burden of disease and budget impact analysis are also needed to support policy-makers with decision-making in health care.

WES for suspected hereditary pediatric cataracts within the SUS could be used as the basis for other ocular genetic diseases, such as retinal dystrophies, which might benefit from molecular diagnosis for currently available gene therapies, such as Voretigene, or for inclusion in ongoing clinical trials [10]. More importantly, this approach can create an opportunity to accelerate the access of patients to genetic testing in routine clinical practice for other rare or inherited diseases and cancers.

Inevitably, genetics will constitute the standard of care and should be available for the community of patients. Accurate information regarding the cost estimation of genetic testing can aid health care policy-makers from middle-income countries in their resource-use assessment for governmental decision-making.

### Electronic supplementary material

Below is the link to the electronic supplementary material.

Supplementary Material 1
