# Supplementary material for: The cost of genetic diagnosis of suspected hereditary pediatric cataracts with whole-exome sequencing from a middle-income country perspective: a mixed costing analysis
Source: J Community Genet. 2024 May 10;15(3):235–47. doi: 10.1007/s12687-024-00708-9 (PMC11217199; doi:10.1007/s12687-024-00708-9)
Supplement: Supplementary file 1 — Supplementary Material 1 [file 12687_2024_708_MOESM1_ESM.docx]

**Supplemental Material – I - Equipment Costs and Distribution of use per step**

**% Equipment Utilization per step**

| **Equipment** | **Unit Cost (BRL)** | **Quantity** | **Total Cost (BRL)** | **Peripheral Blood Collection** | **DNA Extraction** | **Library Preparation** | **NGS** | **Analysis** | | | **Sanger Sequencing** | |
| --- | --- | --- | --- | --- | --- | --- | --- | --- | --- | --- | --- | --- |
| Fluorometer - TF QUBIT Flex | R$ 46.431,00 | 2 | R$ 92.862,00 | 0% | 20% | 20% | 40% | 0% | | | 20% | |
| Spectrophotometer - Nanodrop eight | R$ 37.667,50 | 2 | R$ 75.335,00 | 0% | 50% | 0% | 0% | 0% | | | 50% | |
| Basic Vortex Mixer - TF Vortex | R$ 543,30 | 4 | R$ 2.173,21 | 0% | 14% | 48% | 10% | 0% | | | 29% | |
| Mini Centrifuge - mySPIN™ 12 | R$ 3.500,00 | 2 | R$ 7.000,00 | 0% | 14% | 71% | 14% | 0% | | | 0% | |
| Centrifuge - Eppendorf | R$ 3.652,51 | 2 | R$ 7.305,01 | 0% | 22% | 43% | 9% | 0% | | | 26% | |
| Refrigerator | R$ 2.265,66 | 2 | R$ 4.531,32 | 20% | 20% | 20% | 20% | 0% | | | 20% | |
| Freezer (- 50c) | R$ 35.980,00 | 2 | R$ 71.960,00 | 0% | 25% | 25% | 25% | 0% | | | 25% | |
| Freezer (-4c) | R$ 2.708,00 | 2 | R$ 5.416,00 | 0% | 25% | 25% | 25% | 0% | | | 25% | |
| Thermal Cyclers - TF Applied Biosystems | R$ 52.554,03 | 1 | R$ 52.554,03 | 0% | 0% | 0% | 0% | 0% | | | 100% | |
| Sequencing System - Ilumina NovaSeq6000 | R$ 4.985.125,00 | 1 | R$4.985.125,00 | 0% | 0% | 0% | 100% | | 0% | 0% | |  |
| Computers | R$ 3.500,00 | 44 | R$154.000,00 | 7% | 7% | 7% | 7% | 67% | | | 7% | |
| Thermal Cyclers - Veriti™ 96 | R$ 49.550,00 | 1 | R$ 49.550,00 | 0% | 0% | 0% | 0% | 0% | | | 100% | |
| DNA Extraction - Maxwell RSC | R$ 211.000,00 | 1 | R$211.000,00 | 0% | 0% | 100% | 0% | 0% | | | 0% | |
| Automated pipetting - EpMotion | R$ 861.189,92 | 4 | R$3.444.759,68 | 0% | 0% | 100% | 0% | 0% | | | 0% | |
| Barcode Printer | R$ 1.770,00 | 2 | R$ 3.540,00 | 20% | 20% | 20% | 20% | 0% | | | 20% | |
| Barcode Reader | R$ 714,00 | 2 | R$ 1.428,00 | 20% | 20% | 20% | 20% | 0% | | | 20% | |
| Imaging System - iBright CL1500 | R$ 241.216,00 | 1 | R$241.216,00 | 0% | 0% | 0% | 0% | 0% | | | 100% | |
| Electrophoresis Chamber | R$ 3.258,00 | 1 | R$ 3.258,00 | 0% | 0% | 0% | 0% | 0% | | | 100% | |
| Electrophoresis Power Supply | R$ 3.878,00 | 1 | R$ 3.878,00 | 0% | 0% | 0% | 0% | 0% | | | 100% | |
| Thermal Cyclers - Proflex | R$ 58.392,49 | 2 | R$116.784,98 | 0% | 0% | 25% | 75% | 0% | | | 0% | |
| Pipette 20microl | R$ 550,86 | 6 | R$ 3.470,42 | 0% | 25% | 25% | 25% | 0% | | | 25% | |
| Pipette 100microL | R$ 550,86 | 6 | R$ 3.470,42 | 0% | 25% | 25% | 25% | 0% | | | 25% | |
| Pipette 1000microL | R$ 170,00 | 6 | R$ 1.071,00 | 0% | 25% | 25% | 25% | 0% | | | 25% | |
| Pipette 2 microL | R$ 960,45 | 6 | R$ 6.050,84 | 0% | 25% | 25% | 25% | 0% | | | 25% | |
| Multichannel pippete 12 - 0-10microL | R$ 3.291,66 | 6 | R$ 20.737,46 | 0% | 25% | 25% | 25% | 0% | | | 25% | |

Exchange Rate = 1 USD to 5,1686 BRL

**Supplemental Material – II – Disposable consumables per step**

| **Consumables** | **Quantity per batch** | **Price per Batch (BRL)** | **Unit cost (BRL)** | **Quantity per exam** |
| --- | --- | --- | --- | --- |
| Storage cloud |  | R$ 5,80 | R$ 5,80 | 1 |
| Gas - reference scenario (104km/ month) | 8km/L | R$ 5,56/L | R$ 5,56 | 3,33 |
| Gas - alternative scenario (396 km/month) | 8km/L | R$ 5,56/L | R$ 5,56 | 0,6 |
| **Pheripheral blood collection** |  |  |  |  |
| Gloves | 100 | R$ 45,77 | R$ 0,46 | 4 |
| EDTA tubes 0,5mL | 1 | R$ 13,18 | R$ 13,18 | 1 |
| Vaccum system (needle, tuve and support) | 1 | R$ 8,97 | R$ 8,97 | 1 |
| Gauze | 500 | R$ 18,46 | R$ 0,04 | 2 |
| Alchool 70% | 1L | R$ 7,16 | R$ 0,02 | 1 |
| Micropore | 1 | R$ 2,60 | R$ 2,60 | 2 |
| Isopor box | 1 | R$ 61,33 | R$ 61,33 | 0,03 |
| Dry ice | 1kg | R$ 9,46 | R$ 0,01 | 3 |
| Disposal box/ descarpack | 1 | R$ 11,09 | R$ 11,09 | 0,02 |
| **DNA extraction** |  |  |  |  |
| Gloves | 100 | R$ 45,77 | R$ 0,46 | 6 |
| Tips 20microL | 960 | R$ 1.274,90 | R$ 1,33 | 8 |
| Tips 200microL | 960 | R$ 1.274,90 | R$ 1,33 | 8 |
| Tips 1000 microL | 960 | R$ 1.274,90 | R$ 1,33 | 8 |
| Ethanol 96-100% | 2.5L | R$ 361,87 | R$ 17,37 | 120mL |
| Phosphate Buffered Saline (PBS) | 1L | R$ 731,00 | R$ 0,001 | 100microL |
| Sterile DNase–free microcentrifuge tubes – eppendorf (prepare lysate using digestion buffer and proteinase k) | 500 | R$ 407,18 | R$ 0,81 | 2 |
| PureLink Genomic DNA mini Kit Thermofisher (USA) - KIT step 3 - 250 preparations | 250 | R$ 3.677,12 | R$ 14,71 | 1 |
| 200microL ethanol 96-100% | 2.5L | R$ 361,87 | R$ 0,03 | 200microL |
| Sterile, DNase-free 1.5-mL microcentrifuge tubes for elution | 500 | R$ 407,18 | R$ 0,81 | 2 |
| Sterile water, pH 7.0–8.5 | 100 | R$ 195,25 | R$ 1,95 | 1 |
| Qubit “Labeling & Detection” Invitrogen kit step 3 | 500 | R$ 2.186,60 | R$ 4,37 | 1 |
| Sterile, DNase-free 1.5-mL microcentrifuge tubes (DNA low binding ) | 500 | R$ 407,18 | R$ 0,81 | 3 |
| Sterile water botles | 500 | R$ 2,60 | R$ 0,01 | 1 |
| **Library preparation** |  |  |  |  |
| Gloves | 100 | R$ 45,77 | R$ 0,46 | 8 |
| 96 well titer plate (200ul) | 960 | R$ 430,36 | R$ 0,45 | 1 |
| Illumina® DNA Prep, with Exome 2.0 Plus Enrichment Ref 20077596 | 96 | R$ 66.485,00 | R$ 692,55 | 1 |
| Eppendorf® microtubes | 500 | R$ 69,20 | R$ 0,14 | 4 |
| 96 well titer plate (200ul) | 10 plates | R$ 430,36 | R$ 0,45 | 8 |
| Tips 20microL | 960 | R$ 1.274,90 | R$ 1,33 | 22 |
| Agilent Loading Tips, 1 Pk 5067-5153, | 4.608,00 | R$ 844,13 | R$ 0,18 | 10 |
| Agilent Optical tube strips (8x Strip) 401428 | 5.760 | R$ 399,74 | R$ 0,07 | 3 |
| Agilent Optical tube strip caps (8x Strip) 401425 | 5.760 | R$ 196,18 | R$ 0,03 | 3 |
| Tips for EpMotion - 5075 p50/p300/0030014472/ 0030014430 - Eppendorf | 110 | R$ 4.369,37 | R$ 39,72 | 1 |
| 96 well titer plate deepwell/ P-DW-11-C/ Ciencor (Axygen) | 5 plates | R$ 161,20 | R$ 1,68 | 1 |
| 96 well titer plate (200ul) | 960 | R$ 430,36 | R$ 0,45 | 1 |
| **Next Generation Sequencing** |  |  |  |  |
| Gloves | 100 | R$ 45,77 | R$ 0,46 | 8 |
| NVSEQ 6000 S4 Rgt Kit v1.5 (200cyc) | 240 | R$ 113.272,00 | R$ 471,97 | 1 |
| Ethanol 80% (2ml of water+ 8ml of ETOH 100%) | 20,8 | R$ 361,87 | R$ 17,37 | 200microL |
| Eppendorf plate | 320 | R$ 404,09 | R$ 1,26 | 1 |
| Qubit dsDNA HS Assay Kit Q32854 | 500 | R$ 2.192,40 | R$ 4,38 | 1 |
| 96 Wellplate - Thermo Fisher | 960 | R$ 430,36 | R$ 0,45 | 1 |
| Ethanol 80% (2ml of water+ 8ml of ETOH 100%) | 20,8 | R$ 361,87 | R$ 17,37 | 0,2 |
| Nextera DNA Flex Enrichment Reagents, Illumina DNA Fast Hyb Enrich – Beads and Buffers (20026212)- IDT for Illumina Nextera DNA UD Indexes Set A/B/C/D (96 Indexes, 96 Samples) 20027213, 20027214,20042666, 20042667 | 96 | R$ 4.104,00 | R$ 42,75 | 1 |
| Agencourt AMPure XP Beads ( Beckman Coulter, cA63880,) - Agencourt AMPure XP 60 ml Beckman Coulter A63881 | 414 | R$ 9.900,00 | R$ 23,91 | 1 |
| Midi plate | 320 | R$ 404,09 | R$ 1,26 | 1 |
| Eppendorf plate | 320 | R$ 404,09 | R$ 1,26 | 1 |
| **Analysis** |  |  |  |  |
| HGMDPro | 1 | R$ 1.394,40 | R$ 1.394,40 | 1 |
| CAT MAP | free |  |  |  |
| Annovar | free |  |  |  |
| Varsome | free |  |  |  |
| Franklin | free |  |  |  |
| HPO | free |  |  |  |
| **Sanger sequencing** |  |  |  |  |
| Gloves | 100 | R$ 45,77 | R$ 0,46 | 10 |
| Primer F [10pmol/ µL] 0,5mM Thermo Fisher | 1 | R$ 30,09 | R$ 30,09 | 0,02 |
| Primer R [10pmol/ µL] 0,5mM Thermo Fisher | 1 | R$ 30,09 | R$ 30,09 | 0,02 |
| MgCl2[25Mm] 1,5mM Thermo Fisher | 1 | R$ 147,17 | R$ 147,17 | 0,01 |
| Buffer PCR 5x 1X | 6 | R$ 502,20 | R$ 83,70 | 0,00125 |
| Enzima [5U/ul] 2U/ul | 100 | R$ 684,00 | R$ 6,84 | 0,01 |
| dNTP [25mM] 0,2mM | 0,2 | R$ 447,60 | R$ 447,60 | 0,000125 |
| TAE 1X | 1 | R$ 711,90 | R$ 711,90 | 100mL |
| PCR purification with kit Wizard SV Gel and Clean - Up system | 50 | R$ 774,00 | R$ 15,48 | 1 |
| Styrofoam box for storage and transportation | 1 | R$ 61,63 | R$ 61,63 | 1 |
| 96 well titer plate (200ul) | 960 | R$ 430,36 | R$ 0,45 | 10 |
| Microtubes 1,5mL | 500 | R$ 407,18 | R$ 0,81 | 4 |
| Primers | 1 | R$ 30,09 | R$ 30,09 | 0,04 |
| Water miliQ deionizada e desmineralizada | 1.000.000 | R$ 1.789,65 | R$ 0,0018 | 7,5 |
| Tips 20microL | 960 | R$ 1.274,90 | R$ 1,33 | 24 |
| BigDye v3.1 cylce kit 1 microL - ThermoFisher 4337455 | 100 | R$ 7.789,00 | R$ 77,89 | 1 |
| Buffer Applied Biosystems | 28mL | R$ 1.692,00 | R$ 0,06 | 1,5microL |
| Axygen BF-400-S ou MicroAmp Optical Adesive Film PN:4311971 or Adhesive PCR Film Cat: AB-0558 | 9.600 | R$ 1.659,96 | R$ 0,17 | 10 |
| Corning® Axygen® AxyMats® 96 Round Well Compression Mat for PCR Microplates | 10 | R$ 1.760,00 | R$ 176,00 | 1 |
| Isopropanol 80% MERCK | 1L | R$ 206,12 | R$ 2,06 | 3microL |
| Paper | 1.000 | R$ 20,99 | R$ 0,02 | 2 |
| Ethanol 80% MERCK | 10 | R$ 51,60 | R$ 5,16 | 0,0005 |
| formamida Hi-Di TF 25mL Thermo Fisher PN4311320 | 25.000 | R$ 265,00 | R$ 0,01 | 10 |
| Plate septa Thermo Fisher | 20 | R$ 2.829,57 | R$ 141,48 | 0,083 |
